# Sensitivity of Global Translation to mTOR Inhibition in REN Cells Depends on the Equilibrium between eIF4E and 4E-BP1

**DOI:** 10.1371/journal.pone.0029136

**Published:** 2011-12-22

**Authors:** Stefano Grosso, Elisa Pesce, Daniela Brina, Anne Beugnet, Fabrizio Loreni, Stefano Biffo

**Affiliations:** 1 Molecular Histology and Cell Growth, DIBIT-HSR, Milan, Italy; 2 Dipartimento di Scienze dell'Ambiente e della Vita, University of Eastern Piedmont, Alessandria, Italy; 3 Department of Biology, University Tor Vergata, Roma, Italy; The John Curtin School of Medical Research, Australia

## Abstract

Initiation is the rate-limiting phase of protein synthesis, controlled by signaling pathways regulating the phosphorylation of translation factors. Initiation has three steps, 43S, 48S and 80S formation. 43S formation is repressed by eIF2α phosphorylation. The subsequent steps, 48S and 80S formation are enabled by growth factors. 48S relies on eIF4E-mediated assembly of eIF4F complex; 4E-BPs competitively displace eIF4E from eIF4F. Two pathways control eIF4F: 1) mTORc1 phosphorylates and inactivates 4E-BPs, leading to eIF4F formation; 2) the Ras-Mnk cascade phosphorylates eIF4E. We show that REN and NCI-H28 mesothelioma cells have constitutive activation of both pathways and maximal translation rate, in the absence of exogenous growth factors. Translation is rapidly abrogated by phosphorylation of eIF2α. Surprisingly, pharmacological inhibition of mTORc1 leads to the complete dephosphorylation of downstream targets, without changes in methionine incorporation. In addition, the combined administration of mTORc1 and MAPK/Mnk inhibitors has no additive effect. The inhibition of both mTORc1 and mTORc2 does not affect the metabolic rate. In spite of this, mTORc1 inhibition reduces eIF4F complex formation, and depresses translocation of TOP mRNAs on polysomes. Downregulation of eIF4E and overexpression of 4E-BP1 induce rapamycin sensitivity, suggesting that disruption of eIF4F complex, due to eIF4E modulation, competes with its recycling to ribosomes. These data suggest the existence of a dynamic equilibrium in which eIF4F is not essential for all mRNAs and is not displaced from translated mRNAs, before recycling to the next.

## Introduction

Extracellular signals control the translational machinery, inducing phosphorylation and changes in the activity of translation factors. However, the rapidity by which the translational machinery responds to extracellular signaling, regulating rate and mRNA selection has not been systematically addressed. Initiation of translation is the rate-limiting step of translation, and is controlled by initiation factors (IF) [1], [2]. Three steps are required.

1) Formation of 43S through the recruitment of the ternary complex, formed by eIF2-GTP-tRNA(i)Met, on the small 40S ribosomal subunit. Inhibition of eIF2 activity is mediated by phosphorylation of eIF2α subunit by either one of four eIF2α kinases, PERK, Gcn2, HRK, PKR switched on by several conditions, including ER stress, aminoacid deprivation, lack of heme, double strand RNA [3], [4]. The net result of eIF2α phosphorylation is a block in general initiation. However, specific ORFs, which are silenced in conditions of high levels of ternary complex because they lie downstream of short upstream ORFs (uORFs), are translated upon eIF2α phosphorylation [5].

2) Formation of 48S after the binding of mRNA complexed to eIF4F, and scanning to the AUG start codon. The 5′ end of mRNA binds 43S, mediated by eIF4F. eIF4F is a multiprotein complex: initiation factors eIF4E, eIF4G, and eIF4A assemble on the m^7^Gppp cap structure [6]. 4E-binding proteins (4E-BPs) are inhibitory factors preventing eIF4F formation by binding eIF4E and replacing eIF4G [7]. Growth factor activation controls eIF4F assembly [1]. In response to growth factors, 4E-BPs are hyper-phosphorylated, and released from eIF4E [8], allowing eIF4G recruitment on eIF4E. Although the bulk of translation is cap-dependent and stimulated by eIF4F, some mRNAs do not require eIF4F activity, and can be translated in conditions of impaired translation [9]. Examples are represented by IRES-mediated translation, in which an Internal Ribosome Entry Site mediates the direct attachment of the ribosome in the absence of eIF4F [10], [11].

In principle, eIF4F assembly depends from signaling pathways. Prominently, the phosphoinositide 3-kinase (PI3K) signaling pathway, one of the most frequently deregulated oncogenic pathways, converges on the serine/threonine kinase Akt, and to the mammalian target of rapamycin (mTOR) kinase [12], [13]. The mTOR kinase assembles with either Raptor or Rictor to generate two distinct complexes, mTOR complex 1 and 2 (mTORc1 and mTORc2), respectively. The most known translational regulators phosphorylated by mTORc1, are eIF4E binding proteins 4E-BP1, 4E-BP2, and 4E-BP3 (4E-BPs) and ribosomal protein S6 kinase p70 (S6k1). The result of 4E-BPs phosphorylation is increasing eIF4F activity due to the lack of eIF4E repression. In addition, activation of the Ras pathway leads to the phosphorylation and activation of MAP-interacting kinases-1/2 (Mnk1 or Mnk2), which in turn phosphorylate eIF4E [14], [15]. Although the exact mechanism by which eIF4E phosphorylation affects translation is unclear, it may increase affinity of eIF4E for the mRNA cap structure and for eIF4G. The mTORc1 pathway is efficiently inhibited by rapamycin and its analogues. Recently, others and we described the relative insensitivity of several cancer cell lines and patients to the action of rapalogues; the insensitivity was linked to mutations in the Ras pathway [16]. In line with this, we suggested the presence of rapamycin insensitive translational regulation stimulated by PKC [17] or by adhesion to extracellular matrix [18]. Others reported that rapamycin does not inhibit translation recovery after hypertonic stress [19]. These observations raised the question on how different signaling pathways operate on the translational machinery, whether they are redundant or whether they may be dispensable.

3) The third regulating step of initiation is the formation of elongating 80S, upon recruitment of free 60S subunits and release of eIF2, assisted by eIF5B [20]. Little is understood about regulation of 80S formation. eIF6 plays an important role in maintaining free 60S ribosomal subunits [21], [22], downstream of the growth factor stimulated pathways [23]. eIF6 depletion results in impaired translation, upon insulin stimulation [24].

To further dissect the signaling pathways leading to translational machinery regulation, we screened for cells where the mTORc1 and Ras/ERK pathways were active and studied the effect of their inhibition on translation. We focused on short term treatment with inhibitors, in order to analyze direct effects on the translational machinery. We found that in REN and NCI-H28 cell lines, initiation of translation was insensitive to the treatment with mTOR inhibitor, with exception for translation of TOP mRNAs. Sensitivity to inhibition of the mTORc1 pathway could be reestablished only by 4E-BP1 expression or eIF4E downregulation. These data lead to a model in which the rate of rapamycin sensitivity can be regulated transcriptionally. The lack of effects on global translation by short term inhibition of the main pathways suggests that initiation factors can recycle to a new mRNA efficiently without being regulated by mTORc1 or Ras signaling.

## Results

### Translation of REN cells is growth factors independent and is abrogated by eIF2α phosphorylation

**Figure 1:**
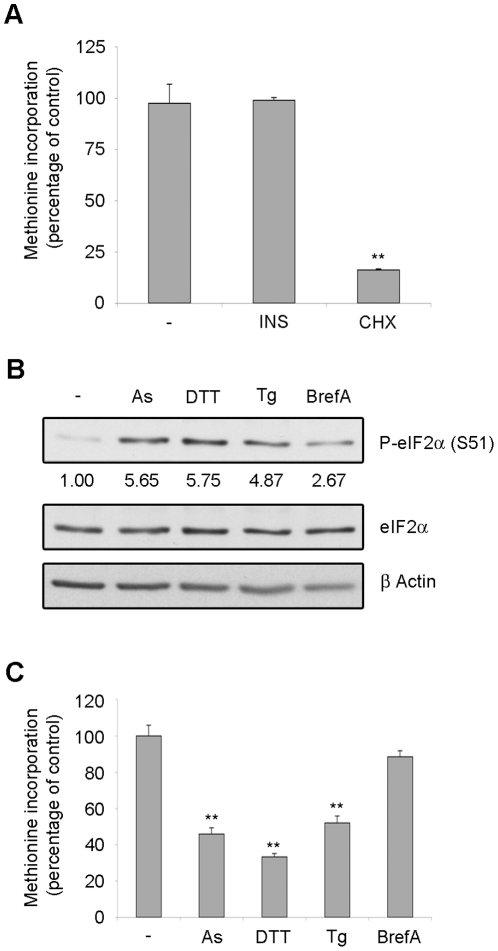
Translation rate is insensitive to exogenous growth factors but sensitive to ER stress. (A) REN cells were starved overnight of growth factors. Cells were treated with 50 nM insulin (INS), with 50 µg/ml cycloheximide (CHX) or left untreated and pulsed with ^35^S-methionine for 45 minutes. Methionine incorporation in newly translated proteins was measured in triplicate. Insulin does not increase translation in REN cells. (B) REN cells were starved overnight of growth factors. Cells were treated with 100 µM arsenite (As), 10 mM dithiothreitol (DTT), 2 µM thapsigargin (Tg) or 10 µg/ml brefeldin A (BrefA) for 45 minutes and total proteins were extracted. Total extracts were analyzed in WB to test eIF2α phosphorylation. Densitometric analysis of phospo-eIF2α normalized to total eIF2α is reported. (C) REN cells were starved overnight of growth factors. Cells were treated as indicated and pulsed with ^35^S-methionine. Methionine incorporation in newly translated proteins was measured in triplicate. Arsenite, dithiothreitol and thapsigargin block translation in REN cells.

We screened for cell lines with constitutively activated mTOR-Akt pathway in order to test the hypothesis that activated pathways lead to deregulated translation, which can be targeted by pharmacological treatment. We found a malignant mesothelioma cell line REN, able to grow under starving conditions that was further characterized. Most mesothelioma cells [25] respond to insulin/IGF-I by increasing protein synthesis, as measured by methionine incorporation. REN cells were serum starved and methionine incorporation was measured in either insulin stimulated or unstimulated cells. Methionine incorporation was not significantly affected by insulin stimulation (Fig. 1A). In starved cells, methionine incorporation was reduced to 10% by cycloheximide treatment, that blocks elongation, indicating that it was due to active protein synthesis (Fig. 1A).

Mesothelioma cells are actively secreting cells [26] and may be susceptible to ER stress. In contrast to the insensitivity to growth factor deprivation, REN cells were highly susceptible to membrane stress. Administration of thapsigargin (Tg) [27], dithiothreitol (DTT), which are ER stress inducing drugs, or arsenite (As) [28] lead to eIF2α phosphorylation (Fig. 1B and Fig. S1A) and block of methionine incorporation (Fig. 1C). Brefeldin A, which inhibits protein transport from ER to Golgi [29] causes a minor eIF2α phosphorylation after short treatment, which is not sufficient to block the translation rate. In these conditions eIF2α phosphorylation does not lead to caspase 3 activation (Fig. S1B).

Taken together, these data indicate that REN cells are highly sensitive to ER stress-induced block of protein synthesis, i.e. 43S formation, but are independent from growth factor stimulation. These data suggest that REN cells have constitutive activation of the growth-factor activated pathways, and translation should be repressed by inhibiting signaling pathways.

### Short-term inhibition of mTOR does not affect initiation and methionine incorporation

**Figure 2:**
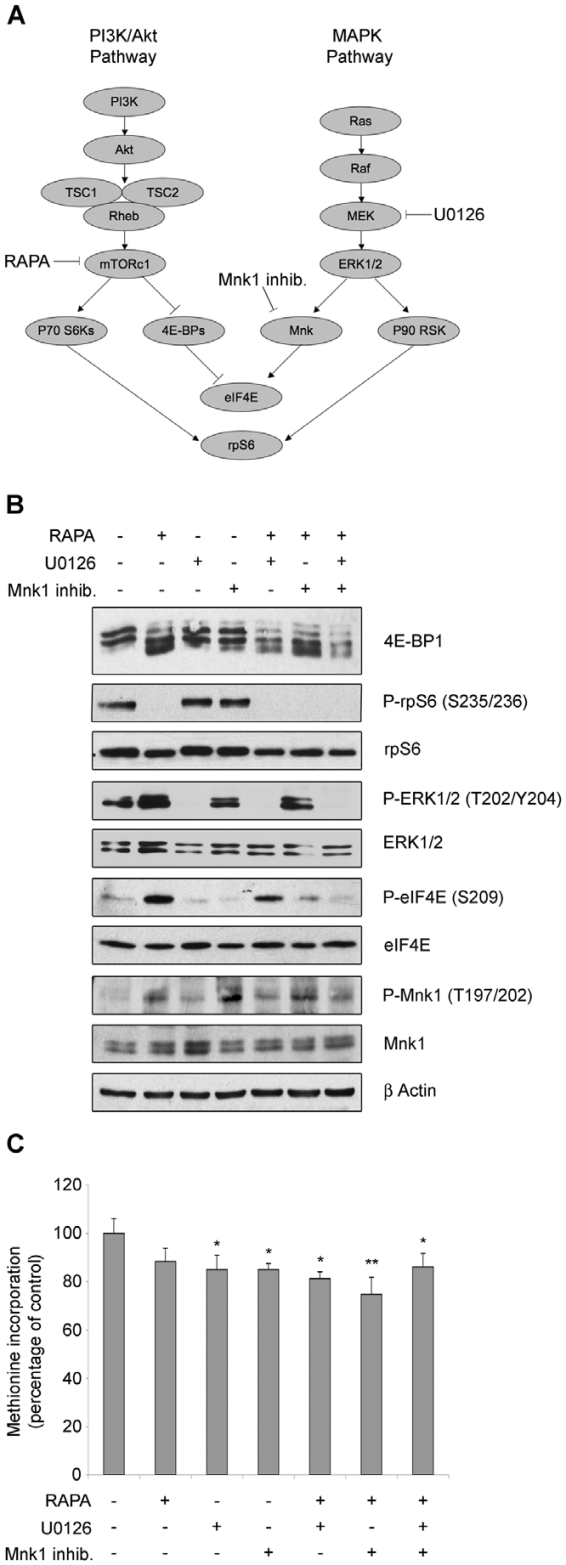
Translation rate is largely insensitive to the efficient inhibition of mTORc1. (A) Scheme of major linear pathways regulating translation. (B) REN cells were treated for 45 minutes with 50 nM rapamycin (RAPA), 10 µM U0126, 5 µM 4-Amino-5-(4-fluoroanilino)-pyrazolo[3,4-d]pyrimidine (Mnk1 inhib.) as indicated and total proteins were extracted. Total extracts were analyzed by WB to test activity of mTORc1, ERK1/2 and Mnk1 signaling. Rapamycin blocks mTORc1 activity, preventing 4E-BP1 and rpS6 phosphorylation. U0126 inhibits MEK, preventing phosphorylation of downstream targets ERK1/2, Mnk1 and eIF4E. Mnk1 inhibitor 4-Amino-5-(4-fluoroanilino)-pyrazolo[3,4-d]pyrimidine blocks eIF4E phosphorylation. (C) REN cells were treated as indicated and pulsed with ^35^S-methionine. Methionine incorporation in newly translated proteins was measured in triplicate. mTOR, ERK1/2, or Mnk1 inactivation minimally reduce translation. Even combination of multiple drugs, in order to switch off multiple signaling pathways, has no additive effect on reducing protein synthesis.

Two main pathways transduce the growth factor stimulation to the translational machinery: PI3K-mTORc1 and Ras-MAPK-Mnk. These two pathways are always deregulated in solid tumors, and converge on the translational machinery (Fig. 2A). We wanted to evaluate if either one, or both of them, can regulate translation in these cells. REN were treated with kinase inhibitors: mTORc1 inhibitor rapamycin, MEK inhibitor U0126, Mnk1 inhibitor 4-Amino-5-(4-fluoroanilino)-pyrazolo[3,4-d]pyrimidine. Analysis of phosphorylation of downstream targets was performed. In basal conditions we found that mTORc1 targets 4E-BP1 and rpS6, Mnk1 target eIF4E, and Ras target ERK1/2 were all phosphorylated. These results are in agreement with the observation that stimulation of REN cells with insulin does not lead to increased methionine incorporation. Treatment with rapamycin resulted in dephosphorylation of 4E-BP1 and rpS6, suggesting the complete switch off of the signaling cascade. U0126 treatment abrogated ERK1/2 phosphorylation downstream of MEK kinase. The inhibition of Mnk1 abrogated phosphorylation of downstream target eIF4E (Fig. 2B). In contrast with the efficient dephosphorylation of all targets after treatment with these drugs, only a minimal drop of translation rate was measured by methionine incorporation (Fig. 2C).

It has been reported that mTORc1 inactivation by rapamycin results in a feedback activation of ERK1/2 [30]. In agreement with this, REN cells treated with rapamycin showed increased eIF4E and ERK1/2 phosphorylation (Fig. 2B). Thus, we hypothesized that the simultaneous inhibition of MAPK and mTORc1, could abrogate phosphorylation of downstream targets and result in a decrease in methionine incorporation. Coadministration of rapamycin and U0126 led to the complete dephosphorylation of ERK1/2, but not of eIF4E. However we did not observe an additive reduction in methionine incorporation (Fig. 2C). p38 MAPK remained phosphorylated with all treatments (not shown) and may account for the residual eIF4E phosphorylation [31].

**Figure 3:**
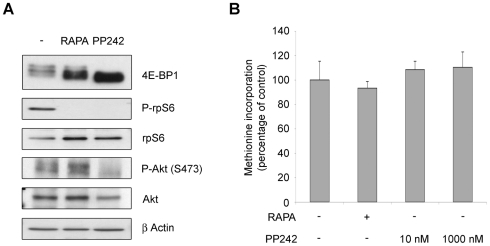
PP242 does not affect translation rate. (A) REN cells were treated with rapamycin or with PP242 and total proteins were extracted. Total extracts were analyzed in WB to test activity of mTORc1 and mTORc2 signaling. PP242 blocks both rpS6 and 4E-BP1 phosphorylation by mTORc1 and Akt phosphorylation by mTORc2. (B) REN cells were treated as indicated and pulsed with ^35^S-methionine. Global translation was measured in triplicate. mTOR kinase inactivation does not reduce translation.

It is established that rapamycin blocks mTORc1 complex, but not mTORc2 complex. mTORc2 phosphorylates Akt, accounting for the activation of a pathway that may impinge on survival and on the translational machinery [32]. mTOR kinase inhibition was performed with PP242, an inhibitor that targets the ATP binding domain, blocking both mTORc1 and mTORc2 signalling [33]. We treated cells with PP242 and tested the phosphorylation of mTORc1 and mTORCc2 downstream effectors. 4E-BP1, rpS6 and Akt were dephosphorylated. (Fig. 3A). Then we measured methionine incorporation in REN cells treated with different concentration of PP242, both at the EC50 of the compound and 100 times more concentrated. Surprisingly, methionine incorporation was unaffected by PP242 treatment (Fig. 3B).

These data indicate that in REN cells translation rates are insensitive to the inhibition of growth factor signaling pathways.

### Inhibition of mTOR changes the turnover of mRNA classes on ribosomes

**Figure 4:**
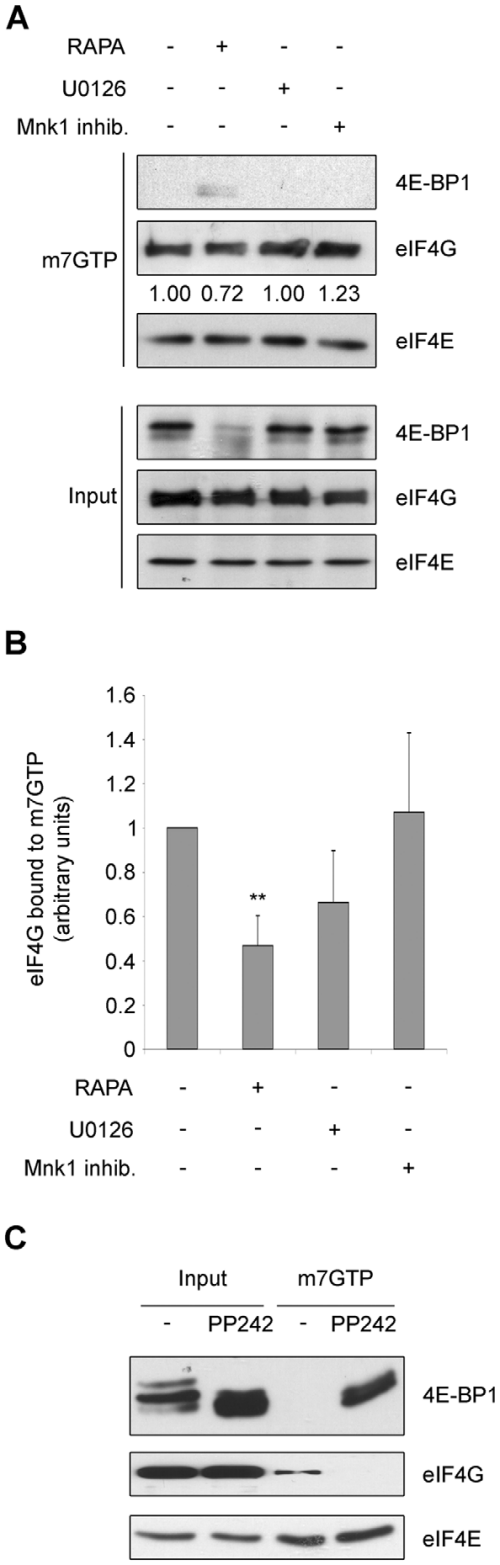
The cap complex formation is inhibited by mTOR inactivation. (A) REN cells were treated as indicated and total proteins were incubated with 7-Methyl GTP-Sepharose beads. Input is 10% of the purification. Cap binding proteins were analyzed by WB with anti-4E-BP1 and with anti-eIF4G. eIF4E shows equal amount of purified proteins. Rapamycin treatment induces 4E-BP1 binding to eIF4E and eIF4G is displaced from the complex. Densitometric analysis of eIF4G levels normalized to eIF4E are reported. (B) Densitometric analysis of eIF4G levels normalized to eIF4E, from 5 independent experiments are reported. (C) REN cells were treated as indicated and total proteins were incubated with 7-Methyl GTP-Sepharose beads. Input is 5% of the purification. Cap binding proteins were analyzed by WB with anti-4E-BP1 and with anti-eIF4G. eIF4E shows equal amount of purified proteins.

A known readout of growth factor stimulation is the assembly of the cap complex, which is dependent on mTOR signaling. Data in Figure 4A show that mTORc1 inhibition by rapamycin causes an enrichment of 4E-BP1 bound to eIF4E which displaces the binding to eIF4G i.e. inhibition of cap complex formation. The effect of Mnk/MAPK inhibition was subtle; U0126 did not affect 4E-BP1 binding to eIF4E, but caused a minimal decrease of eIF4G binding (Fig. 4B). mTORc1 and mTORc2 inhibition by PP242 strongly affects the cap complex assembly of initiation factors (Fig. 4C). It follows that i) since this disruption is not accompanied by a decrease in methionine incorporation, other factors may be rate-limiting, ii) at least some mRNAs may be affected by cap complex inhibition, but not by MAPK inhibition.

**Figure 5:**
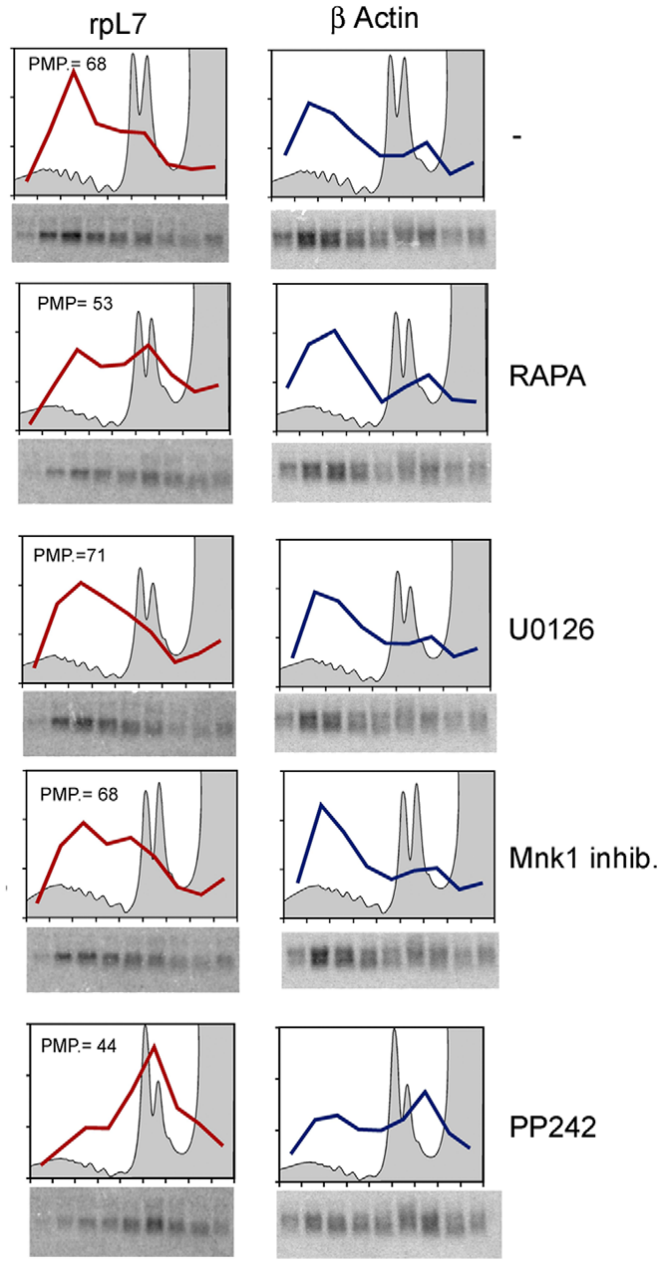
TOP mRNA translation is inhibited by mTOR inactivation. REN cells were treated as indicated and total extracts were loaded on sucrose gradient. Polysomal profiles were performed, fractions from the gradient were collected and RNA was precipitated. Northern Blot analysis of rpL7 (TOP) and β-Actin mRNAs were performed. Polysomal profiles confirm that blocking mTOR and MAPK signaling has no effect on global translation. Rapamycin and PP242 displace TOP messengers from polysomes. PMP indicates the percentage of rpL7 mRNA associated to polysomes.

Thus, we asked whether inhibition of mTORc1 and MAPK signaling affected translation of specific mRNAs. Polysomal profiles of cells treated with inhibitors showed small changes in 80S/polysomal ratio, confirming the minimal reduction of initiation when the pathways are blocked (Fig. 5). Fraction from polysomes were then collected and analyzed by Northern Blot to analyze the distribution of translated mRNAs. We focused on TOP mRNAs that encode for ribosomal proteins and a number of translation factors, because they are strongly dependent from mTOR signaling [34]. Briefly, we found a slight decrease in polysomal distribution of TOP mRNA in cells treated with rapamycin and PP242 (Fig. 5), suggesting that the translation of this class of mRNAs is specifically sensitive to 4E-BP1 bound to eIF4E, disrupting the cap complex. The levels of TOP mRNA translation remained however fairly high, indicating that most mRNAs escaped the inhibition of eIF4F formation. Taken together, data suggest that even upon inhibition of 4E-BP1 phosphorylation, sufficient eIF4E is present in the cell to guarantee proper initiation. However, the composition of translated mRNAs changes upon inhibition of the mTOR pathway.

### Restoring rapamycin sensitivity through transcriptional modulation of eIF4F factors

**Figure 6:**
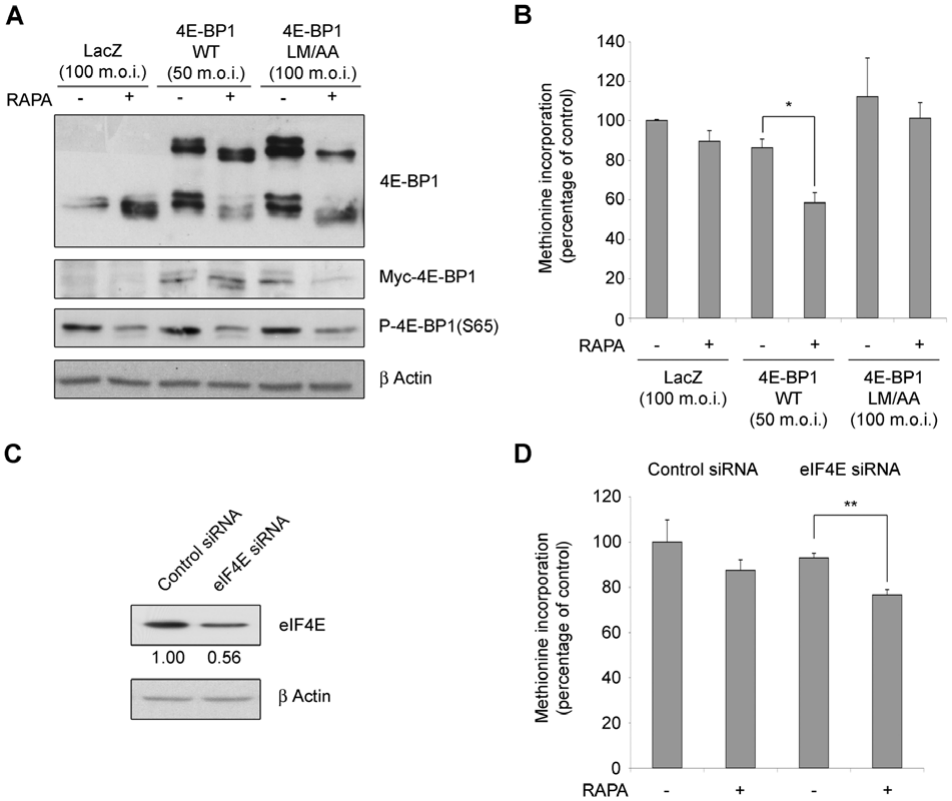
4E-BP1 overexpression or eIF4E reduction restore rapamycin sensitivity. (A) REN cells were infected with myc tagged-4E-BP1 WT, LM/AA (unable to bind eIF4E) or with control adenovirus with the indicated Multiple of Infection (m.o.i.). WT and LM/AA HA-4E-BP1 expression was confirmed by WB analysis of the myc tag. Exogenous 4E-BP1 is expressed as the same level as the endogenous protein. Serine 65 phosphorilation of 4E-BP1 is reduced by rapamycin treatment, as expected. (B) Infected cells were treated with rapamycin or left untreated and pulsed with ^35^S-methionine. Methionine incorporation in newly translated proteins was measured in triplicate. WT 4E-BP1 overexpression restores the rapamycin sensitivity in REN cells. (C) REN cells were transfected with eIF4E siRNA or control. After 48 hrs, total protein were analyzed by WB in order to measure eIF4E downregulation. Densitometric analysis of eIF4E level normalized to β-Actin is reported. (D) REN cells were transfected with eIF4E siRNA or control. Cells were treated with rapamycin and the rate of translation was measured. Rapamycin impairs translation where eIF4E level is reduced.

We then asked what is the rate-limiting event of cap dependent translation of REN cells. Thus, we formulated the hypothesis that 4E-BP1 is not sufficient to sequestrate eIF4E. REN cells were infected with adenovirus overexpressing 4E-BP1 wt, 4E-BP1 LM/AA, protein mutant unable to bind eIF4E [35], or adenoviral construct alone. The levels of overexpressed proteins were detected by WB (Fig. 6A). Then we analyzed the methionine incorporation both in basal conditions and upon rapamycin administration. We found that the level of translation in cells overexpressing 4E-BP1 wt, was reduced after rapamycin treatment. Control adenovirus infected cells, cells overexpressing 4E-BP1 LM/AA or cells overexpressing rapamycin insensitive mutation (4Ala) [36] were not affected by rapamycin treatment (Fig. 6B and Fig. S2A). The overexpression of 4E-BP1 wt or mutated did not affect caspase 3 activation (Fig. S2B).

Next, we downregulated eIF4E, by interfering with specific siRNA. Downregulation was confirmed by western blot (Fig. 6C and Fig. S2C and D) and rapamycin sensitive translation was monitored. mTOR inhibition induces a reduction of global translation in cells with lower eIF4E levels (Fig. 6D). Caspase 3 activation was not affected by eIF4E levels (Fig. S2D).

**Figure 7:**
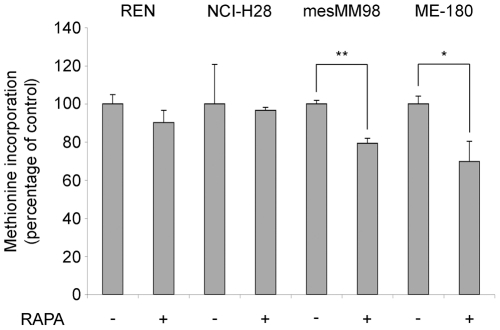
Rapamycin treatment has no effect on global protein synthesis in two epithelial malignant mesothelioma cell lines. REN (epithelial malignant mesothelioma), NCI-H28 (epithelial malignant mesothelioma), mesMM98 (sarcomatoid malignant mesothelioma) and ME-180 (cervical carcinoma) cells were treated with rapamycin and pulsed with ^35^S-methionine. Global translation was measured in triplicate, normalized on total protein content. The level of translation of untreated cells is set to 100%. Rapamycin reduces translation in mesMM98 and ME-180 cell lines, but it is ineffective on REN and NCI-H28.

**Figure 8:**
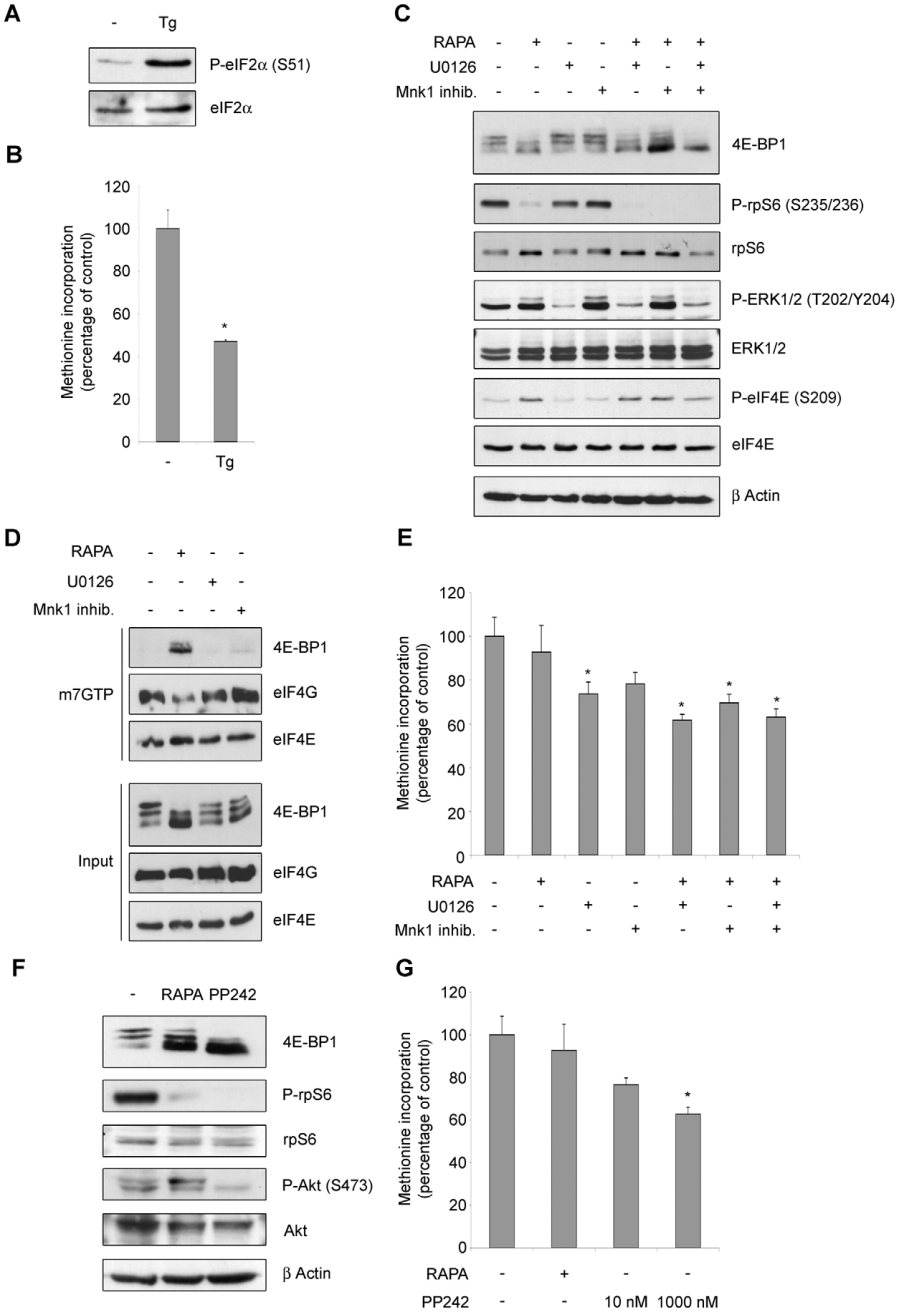
In malignant mesothelioma cell line NCI-H28, mTOR inhibition blocks the formation of the cap complex, without affecting the ongoing translation. (A) NCI-H28 cells were treated with 2 µM thapsigargin (Tg) and total proteins were extracted. Extracts were analyzed by WB to test eIF2α phosphorylation. (B) NCI-H28 cells were treated as indicated and for 45 minutes and pulsed with ^35^S-methionine. Methionine incorporation in newly translated proteins was measured in triplicate. Thapsigargin blocks translation in NCI-H28 cells (C) Cells were treated for 45 minutes with 50 nM rapamycin (RAPA), 10 µM U0126, 5 µM 4-Amino-5-(4-fluoroanilino)-pyrazolo[3,4-d]pyrimidine (Mnk1 inhib.) as indicated and total proteins were extracted. Total extracts were analyzed by WB to test activity of mTORc1, ERK1/2 and Mnk1 signaling. Rapamycin blocks mTORc1 activity. (D) NCI-H28 cells were treated as indicated and total proteins were incubated with 7-Methyl GTP-Sepharose beads. Input is 10% of the purification. Cap binding proteins were analyzed by WB with anti-4E-BP1 and with anti-eIF4G. eIF4E shows equal amount of purified proteins. (E) Cells were treated as indicated and pulsed with ^35^S-methionine. Methionine incorporation in newly translated proteins was measured in triplicate. mTORc1 inactivation does not reduce translation. (F) Cells were treated with rapamycin or with PP242 and total proteins were extracted. Extracts were analyzed by WB to test activity of mTORc1 and mTORc2 signaling. PP242 blocks both rpS6 and 4E-BP1 phosphorylation by mTORc1 and Akt phosphorylation by mTORc2. (G) Cells were treated as indicated and pulsed with ^35^S-methionine. Global translation was measured in triplicate. mTOR kinase inactivation reduces translation only at 1 µM concentration.

So far we have shown that REN cells have an impairment of cap complex formation, but not global reduction of translation, upon mTOR inhibition. Next we evaluated whether this phenomenon is present in other cells. Global translation of ME-180 cells, that have constitutively activated PI3K [37], is sensitive to rapamycin inhibition ([16] and Fig. 7). Similarly, in mesMM98, that have less expression of eIF4E as compared to REN cells (Fig. S3), protein synthesis is sensitive to rapamycin treatment. It has been reported that p53 can downregulate the level of 4E-BP1 and eIF4G [38] and transcriptionally reduce the level of c-myc [39], thus we selected a mesothelioma cell line (NCI-H28) with wt p53 [40]. Surprisingly NCI-H28 cells were not sensitive to rapamycin inhibition of translation (Fig. 7 and 8E). Thus we focused on the characterization of the pathways regulating translation in these cells. First, eIF2α phosphorylation induced by ER stress, caused inhibition of translation (Fig. 8A and B). Next, the pharmacological block of mTORc1, MEK and Mnk1 caused the dephosphorylation of downstream targets (Fig. 8C). However, as for the REN cells, also in NCI-H28 the complete block of the mTORc1 pathway impaired the cap complex assembly (Fig. 8D), without any significative effect on the global translation (Fig. 8E). Even the administration of PP242 did not reduce translation at a significative level (Fig. 8F and G).

Here we conclude that cap complex inhibition does not match the global translation, and that global translation is not necessarily reduced by mTOR inhibition in all cellular types.

## Discussion

We show that in the absence of 4E-BP1 overexpression or eIF4E downregulation, the efficient pharmacological inhibition of mTOR pathway does not change, in short term, the efficiency of the translational apparatus. These data indicate that part of the regulation of translation rate, at least in REN cells, may occur transcriptionally through the abundance of eIF4E. In addition, rapamycin does not depress translation, in spite of reducing cap complex formation. It follows that, *in vivo*, at steady-state recycling of eIF4E from a processed mRNA to a new preinitiation complex is either kinetically or sterically favored respect to binding of eIF4E to 4E-BP.

Translational control is becoming an attractive target in cancer cells [41]. However, we need to further understand the complex ways by which the process of transformation can perturb the translational machinery. REN mesothelioma cells, here characterized, are able to survive and proliferate under growth factor and matrix deprivation, giving rise to metastatic cancer in experimental models [26], [42]. We characterized translational control in REN mesothelioma cells in the absence of exogenous stimulation, in order to establish the relevance of endogenous pathways converging on translation. Malignant mesothelioma is a slow growing tumor resistant to chemotherapy [43]. Here, we report that the metabolic rate of REN cells is not only independent from exogenous growth factor activation, but also largely insensitive to the inhibition of both mTOR and MAPK pathways, suggesting that it is controlled by transcriptional activation of the ribosomal machinery. We observed similar results in NCI-H28, but not in mesMM98 mesothelioma cells.

The transcriptional regulation of initiation factors is an emerging concept, that is becoming relevant in cancer research [44]. Myc is known to regulate the translational machinery [45], [46] and, specifically, it regulates eIF4E and eIF4G levels [47], [48]. Myc also stimulates the output of Pol I [49] and Pol III [50], resulting in the increase of rRNA and tRNA expression. It remains to be established if also 4E-BP1 is under the control of Myc oncogene. REN cells express more c-myc than mesMM98 in line with their rapamycin insensitivity. It has to be established with future work if a causative relation between c-myc expression and eIF4E exists in mesothelioma cells. P53 has been reported to regulate eIF4G and 4E-BP1 [39]. We could not establish a relationship between p53 and rapamycin sensitivity. A more intriguing possibility exists: it was recently shown that mTOR inactivation in the heart led to 4E-BP upregulation [51]. If this is a general case, one contribution of the constitutively active PI3K-mTOR pathway on the translational control, is the stimulation of eIF4F formation by the transcriptional repression of one key player, 4E-BPs, through yet unknown mechanisms. Thus, in cancer cells, cap complex activation may result from three separate routes: inactivation of 4E-BPs by phosphorylation, upregulation of eIF4E and transcriptional downregulation of 4E-BP1. These three elements may not be exclusive.

The marginal effect of the inhibition of signaling pathways on the translational machinery can reflect the fact that we employed short-term treatments. This led to an interesting observation: the temporal disjunction between the efficient inhibition of the pathway and the effect on translational machinery. First, we found that in REN cells rapamycin treatment blocked mTOR dependent assembly of cap complex, without a significative reduction of metabolic rate. Moreover, when we blocked TOR kinase activity with PP242, leading to a complete inhibition of both mTORc1 and mTORc2, even at 100 µM, where PP242 has been reported to aspecifically block PI3K/Akt pathway [33], the global rate of translation was not affected, after short term treatment. We previously found that the sensitivity of the translational apparatus to pharmacological inhibition with Everolimus, a rapamycin analogue, was linked to mutations in the Ras pathway [16]. The MAPK-ERK signaling pathway, downstream of Ras, acts on the initiation of translation, at least at three levels: Mnk1/2 phosphorylate eIF4E at Ser 209, and this event contributes to malignancy [52]; rpS6 is phosphorylated by p90 Ribosomal S6 Kinases [53]; 4E-BP1 has rapamycin insensitive, serum dependent phosphorylation sites [54]. Even if ERK1/2 was constitutively activated in REN cells, the inhibition of either this pathway trough U0126 drug or Mnk1 inhibition, had a modest role on the general level of translation. In addition, the combination of multiple inhibiting drugs was not effective. Finally, when we performed long-term treatment with rapamycin, we were able to reduce cap dependent translation (Grosso, unpublished data). Taken together, these observations are consistent with two interpretations, which are not mutually exclusive: 1) the kinetics of inhibition of eIF4E by 4E-BP1 *in vivo*, is delayed, indicating that eIF4E actively engaged in eIF4F, recycles to the mRNA, rather than binding to free 4E-BPs. This interpretation is backed up by the observation that increasing 4E-BP1 or lowering eIF4E results in a more rapid inhibition of translation by rapamycin; 2) in principle, oncogenic activation by Ras, PI3K or Myc may alter by a transcriptional mechanism the sensitivity of the translational machinery to pharmacological inhibition.

An early event observed after rapamycin treatment is the decrease of translation of TOP mRNAs. This indicates that TOP mRNA translation is highly sensitive to cap complex inhibition as shown in several laboratories (reviewed in [34]). The fact that at the same time the metabolic rate is not yet altered can be due to two reasons: 1) the reduction in TOP mRNA translation is marginal with respect to global protein synthesis and below the sensistivity of our assay, 2) in early phases of inhibition, TOP mRNAs are competitively replaced by other classes of mRNAs that have a reduced requirement for the cap complex. It remains to be established which are the mRNAs that replace TOP on the translating ribosomes. Attractive candidates are IRES containing mRNA that can be translated independently from eIF4F complex [55]. Whatever the mechanism at play, these data strengthen the importance of the regulation of the translational machinery in cell cycle progression, since a continuous feed from the growth factor signaling pathway is required for the synthesis of new ribosomes.

In consideration of the data that we presented, showing that inhibition of signaling pathways may not always affect translation of cancer cells, it may be more fruitful to directly inhibit the association of initiation factors. Recently, it was reported that 4EGI-1, a compound that disrupts eIF4E/eIF4G association, inhibits cap-dependent translation [56]. In this context, also hippuristanol has been reported to block eIF4A helicase activity reducing the translation of structured mRNAs [57], and ribavirin affects the eIF4E localization in the cytoplasm [58], without acting as mimic of mRNA cap structure [59]. It will be interesting to investigate whether REN cells are also insensitive to these compounds.

## Materials and Methods

### Cell Lines and Culture

The following human Malignant Mesothelioma cell lines were used: REN cells, inflammatory epithelial subtype [60]; NCI-H28 (CRL5820), epithelial subtype was purchased from the American Type Culture Collection (ATCC); MesMM98 cells, established from pleural effusion of a sarcomatous mesothelioma [61]. ME-180 cells from endometrial cancer, were purchased from the American Type Culture Collection (ATCC). REN and mesMM98 cells were cultured in Dulbecco's modified Eagle's medium (DMEM); NCI-H28 and ME-180 cells were cultured in RPMI1640. Media were purchased by Lonza, and supplemented with 10% fetal bovine serum (FBS) (from Euroclone) and 1% glutamine and antibiotic mixture (from Gibco).

### Drugs and Reagents

Insulin (human, recombinant), cycloheximide, MEK inhibitor U0126 and mTOR inhibitor PP242 were from Sigma. RNasin® was from Promega. Mnk1 inhibitor 4-Amino-5-(4-fluoroanilino)-pyrazolo[3,4-d]pyrimidine and mTORc1 inhibitor rapamycin were purchased from Calbiochem. All other chemicals were from Sigma.

### Methionine Labeling

Cells were pulsed with 33 µCi per plate of Promix ^35^S-labeled methionine (PerkinElmer Life Sciences) for 45 min, in the tested conditions. Cells were lysed in 50 µl of RIPA buffer without SDS (10 mM Tris–HCl, pH 7.5, 1% Na-deoxycholate, 1% Triton X-100, 150 mM NaCl, 1 mM EDTA, protease inhibitor cocktail from Sigma). The lysate was cleared by centrifugation. Aliquots of 10 µl of extracts were TCA-precipitated and counted with scintillation fluid in a β-counter (Packard). Values were normalized on sample protein content by the bicinchoninic acid (BCA) protein assay (Pierce). Each sample was performed in triplicate and expressed as mean ± SEM. Statistical analysis was performed with T Student test. * is P<0.05. ** is P<0.01.

### Western blotting

Cells were lysed with buffer containing 10 mM NaCl, 10 mM MgCl_2_, 10 mM Tris–HCl, pH 7.3, 1% Triton X-100, 1% Na-deoxycholate, 1 mM DTT, 5 mM NaF, 2 mM Na_3_VO_4_, 40 U/ml RNasin® (Promega), protease inhibitor cocktail. The crude cell extract was clarified at 4°C at 15,000*g* for 10 min and the amount of protein in the supernatant was quantified by the bicinchoninic acid (BCA) protein assay. Equal amount of proteins was analyzed. Extracts were resolved on SDS–PAGE, transferred to Immobilon-P membranes (Millipore), and probed with appropriate antibodies. The following antibodies were used: rabbit anti-phospho-eIF2α (Ser 51), anti-eIF2α, anti-phospho-4E-BP1 (Ser 65), anti-4E-BP1, anti-phospho-rpS6 (Ser 235/236), anti-rpS6, anti-phospho-p44/42 MAPK (ERK1/2) (Thr 202/Tyr204), anti-p44/42 MAPK (ERK1/2), anti-phospho-eIF4E (Ser 209), anti-eIF4E, anti-phospho-Mnk1 (Thr 197/202), anti-Mnk1, anti-phospho-Akt (Ser 473), anti-Akt, anti-eIF4G and anti-c-myc from Cell Signaling; mouse anti-β Actin from Sigma; mouse anti Caspase 3 from Alexis. Densitometric analysis was performed by ImageQuant 5.0 software (Molecular Dynamics).

### m^7^GTP Cap Column Pull-Down

Cap column pull-down was performed on 400 µg of total extracts as described [62].

### Polysomal Profiles and Northern Blot

Polysomal profiles were performed as previously described [63], with some modifications. . Cells (1–2×10^6^) that had been washed once with phosphate-buffered saline buffer (150 mM NaCl, 2.7 mM KCl, 8 mM NaH_2_PO_4_ and 1.4 mM K_2_PO_4_), were lysed directly on the plate with 300 µl of lysis buffer (10 mM NaCl, 10 mM MgCl_2_, 10 mM Tris-HCl pH 7.5, 1% triton-X100, 1% sodiun deoxycholate, 36 U/ml RNase inhibitor, 1 mM dithiothreitol) and transferred into a microcentrifuge tube. After 5 min of incubation in ice with occasional vortexing, the lysate was centrifuged for 10 min at 10,000 rpm (15,000 g) at 4°C. The supernatant was frozen in liquid nitrogen and stored at −70°C to be analyzed later, or immediately layered in a 15–50% (w/v) sucrose gradient containing 30 mM Tris-HCl pH 7.5, 100 mM NaCl and 10 mM MgCl_2_, and centrifuged in a Beckman SW41 rotor for 110 min at 37,000 rpm (170,000 g).

Analysis of the polysome/mRNP distribution of mRNAs were performed essentially as previously described [64]. In particular, polysomal fractions were collected while monitoring the optical density at 254 nm and total RNA was extracted from each fraction by the proteinase K method. For Northern analysis, RNA was fractionated on formaldehyde-agarose gels and transferred to GeneScreen Plus membrane (PerkinElmer Life Sciences). Northern blotting was performed essentially as recommended by the manufacturer. Radioactive probes were prepared by the random primer technique using gel purified cDNA fragments. Quantitation of Northern blots was performed using a PhosphorImager and the Image-Quant software (Amersham).

### Adenoviral and retroviral infections

Adenovirus: Lac Z control, 4E-BP1 WT and 4E-BP1 LM/AA, were a kind gift of Pr C. Proud. Virus stock were amplified and titer were determinated using Adeno-X TM rapid titer kit (ref PT3651-2; Clontech). Multiplication of infection (M.O.I) of 50 for 4E-BP1 WT and of 100 for 4E-BP1 LM/AA and control LacZ were used (as reported in [35]). Retrovirus: pBABE-puro, 4E-BP1 WT and 4E-BP1 4Ala were kind gift of Pr N. Sonenberg. Infection was carried out as previously described [36].

### siRNA (small interfering RNA) transfection

The siRNA targeted to human eIF4E was purchased from Ambion. The sequence is as follows: siRNA ID #4578,5′-GGUAUACAAGGAAAGGUUAtt-3′ (sense) and 5′-UAACCUUUCCUUGUAUACCct-3′ (antisense). Identical not shown results were obtained with siRNA ID #4577, 5′-GCUAAUUACAUUGAACAAAtt-3′ (sense) and 5′-UUUGUUCAAUGUAAUUAGCca-3′ (antisense). The negative control siRNA was purchased from Ambion (negative control siRNA #1).

REN cells (1.5×10^5^) were seeded per plate (35 mm diameter) the day before transfection. Cells were transfected with 20 nM oligonucleotide using N-TER™ Nanoparticle siRNA Transfection System (Sigma) according to manufacturer protocol. Protein expression was assessed by Western blotting at 48 h post-transfection and densitometric analysis was performed. A metabolic assay was carried out at 48 h post-transfection as described above.

## Supporting Information

Figure S1 **ER stress inducing drugs do not cause Caspase 3 activation at the analyzed time point.** (A) Densitometric analysis of phospho-eIF2α normalized to total eIF2α after thapsigargin (Tg) treatment is reported from four independent experiments. (B) Total extracts from figure 1B were analyzed in WB to test activation of Caspase 3. On the left panel, a positive control for activated caspase 3 is shown.
(TIF)

Figure S2 **ER stress inducing drugs do not cause Caspase 3 activation at the analyzed timepoint.** (A) REN cells were infected with HA-4E-BP1 WT, 4Ala (unable to be phosphorylated) or with empty control retrovirus pBABE. Infected cells were treated with rapamycin and pulsed with ^35^S-methionine. Methionine incorporation in newly translated proteins was measured in triplicate. 4Ala-4E-BP1 transfected cells are unable to sense the rapamycin treatment. (B) REN cells extracts from figure 6A were analyzed in WB to test Caspase 3 activation. (C) REN cells were transfected with eIF4E siRNA at 10 nM or 20 nM concentrations. After 48 hrs, total proteins were analyzed by WB in order to measure eIF4E downregulation. A good downregulation of eIF4E protein was obtained at 50 nM siRNA concentration. (D) REN cells were transfected with eIF4E siRNA or control. After 48 hrs, cells were treated with rapamycin and total proteins were analyzed by WB to test eIF4E reduction and Caspase 3 activation.
(TIF)

Figure S3 **C-Myc and initiation factors protein expression in REN and mesMM98 cell lines.** Total protein extract from malignant mesothelioma cell lines REN and mesMM98 were analyzed with WB for eIF4E, eIF4G, 4E-BP1 and c-Myc protein expression. Densitometric analysis of eIF4E, eIF4G, 4E-BP1 and c-Myc levels normalized to β-Actin are reported. REN cell express more c-Myc and eIF4E proteins.
(TIF)
