# Supplementary figures and images for: Sensitivity of Global Translation to mTOR Inhibition in REN Cells Depends on the Equilibrium between eIF4E and 4E-BP1
Source: PLoS One. 2011 Dec 22;6(12):e29136. doi: 10.1371/journal.pone.0029136 (PMC3245250; doi:10.1371/journal.pone.0029136)

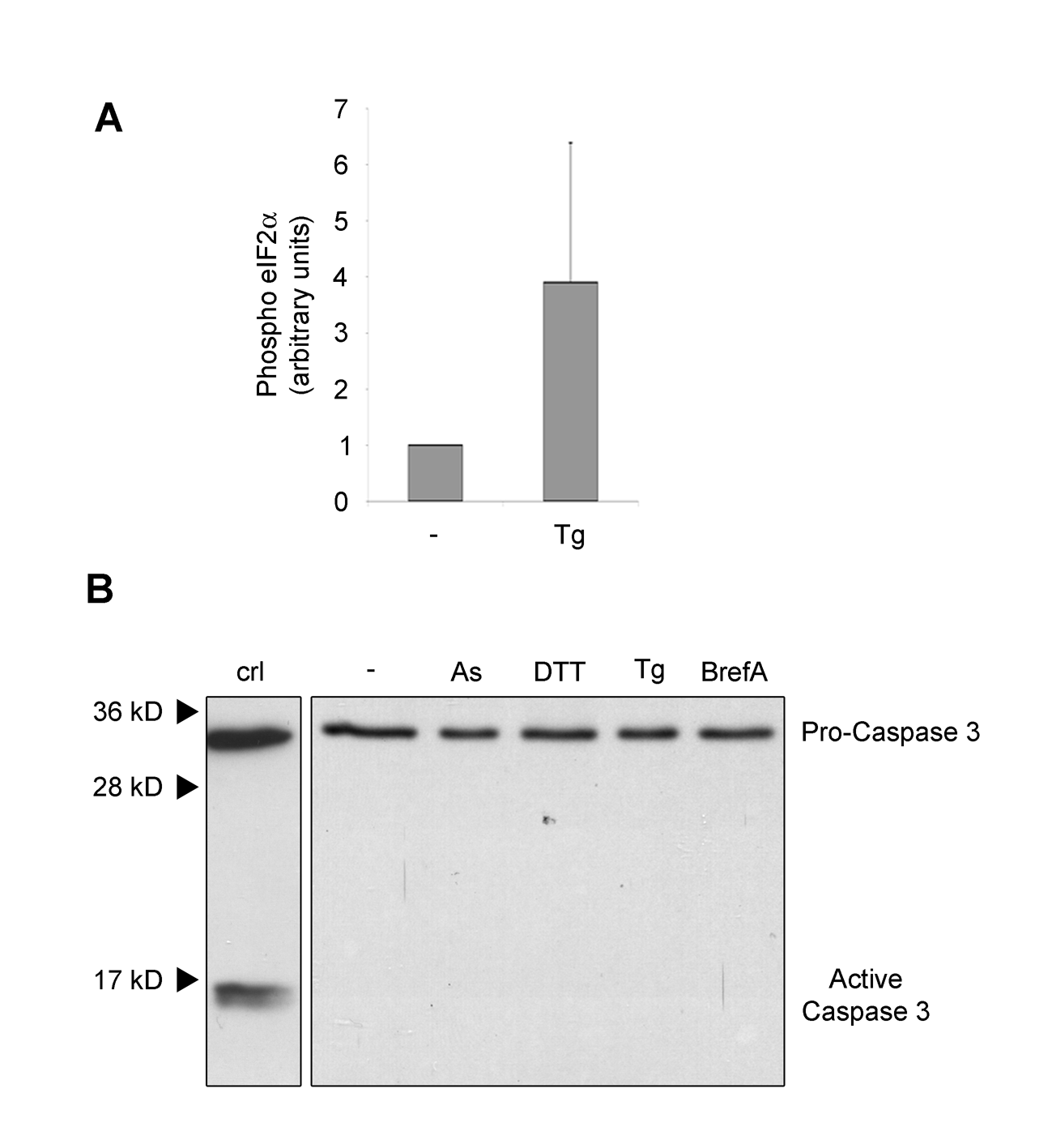

Supplement: Figure S1 — ER stress inducing drugs do not cause Caspase 3 activation at the analyzed time point. (A) Densitometric analysis of phospho-eIF2α normalized to total eIF2α after thapsigargin (Tg) treatment is reported from four independent experiments. (B) Total extracts from figure 1B were analyzed in WB to test activation of Caspase 3. On the left panel, a positive control for activated caspase 3 is shown. (TIF) [file pone.0029136.s001.tif]

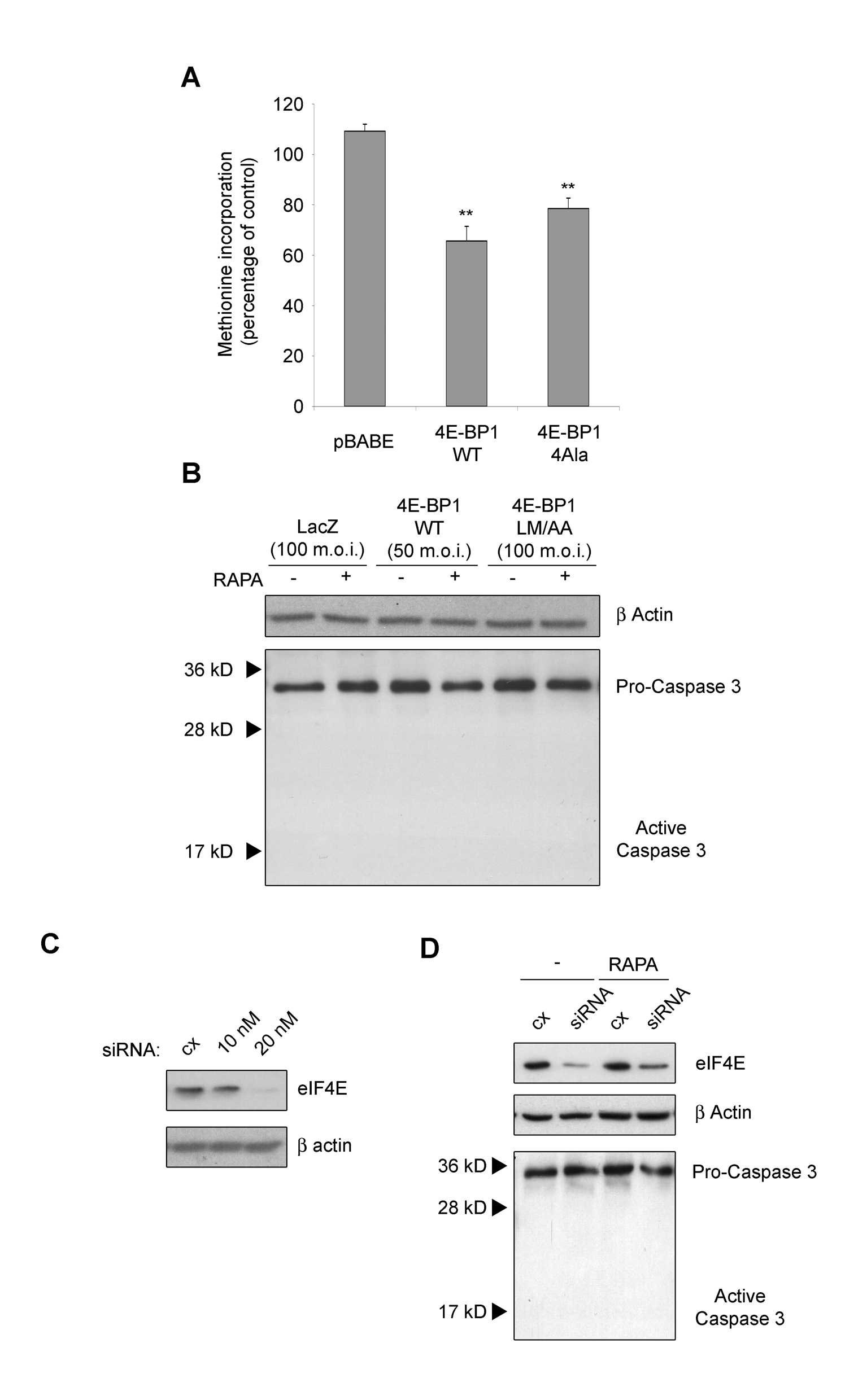

Supplement: Figure S2 — ER stress inducing drugs do not cause Caspase 3 activation at the analyzed timepoint. (A) REN cells were infected with HA-4E-BP1 WT, 4Ala (unable to be phosphorylated) or with empty control retrovirus pBABE. Infected cells were treated with rapamycin and pulsed with 35S-methionine. Methionine incorporation in newly translated proteins was measured in triplicate. 4Ala-4E-BP1 transfected cells are unable to sense the rapamycin treatment. (B) REN cells extracts from figure 6A were analyzed in WB to test Caspase 3 activation. (C) REN cells were transfected with eIF4E siRNA at 10 nM or 20 nM concentrations. After 48 hrs, total proteins were analyzed by WB in order to measure eIF4E downregulation. A good downregulation of eIF4E protein was obtained at 50 nM siRNA concentration. (D) REN cells were transfected with eIF4E siRNA or control. After 48 hrs, cells were treated with rapamycin and total proteins were analyzed by WB to test eIF4E reduction and Caspase 3 activation. (TIF) [file pone.0029136.s002.tif]

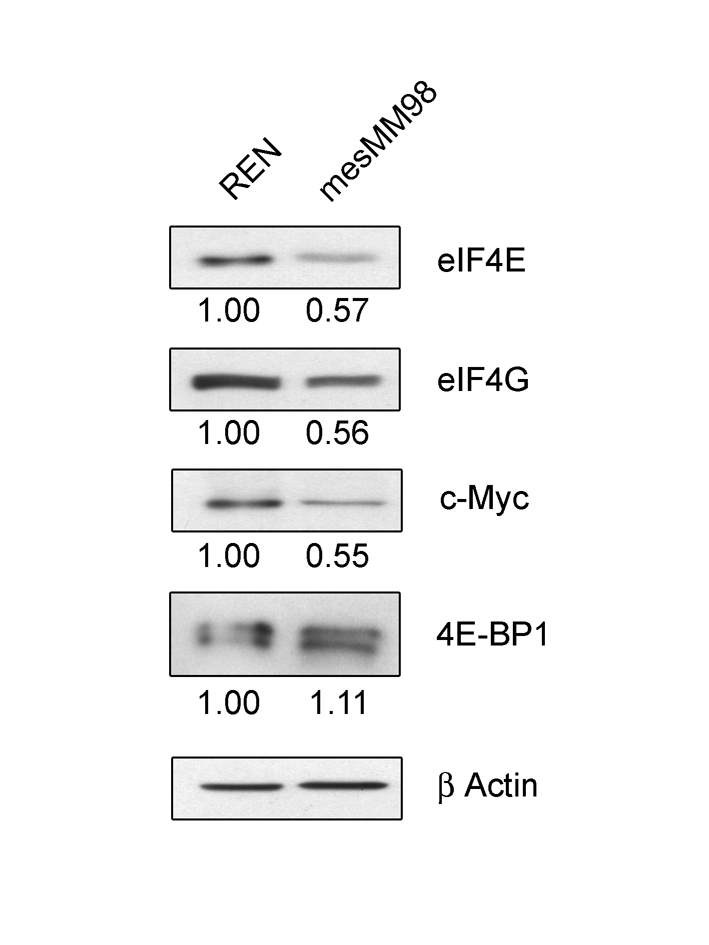

Supplement: Figure S3 — C-Myc and initiation factors protein expression in REN and mesMM98 cell lines. Total protein extract from malignant mesothelioma cell lines REN and mesMM98 were analyzed with WB for eIF4E, eIF4G, 4E-BP1 and c-Myc protein expression. Densitometric analysis of eIF4E, eIF4G, 4E-BP1 and c-Myc levels normalized to β-Actin are reported. REN cell express more c-Myc and eIF4E proteins. (TIF) [file pone.0029136.s003.tif]
